# A Systems Approach to Radiation-Induced Cardiopulmonary Toxicity—A Narrative Literature Review Focusing on the Interdependence of the Heart and the Lung in Thoracic Radiotherapy

**DOI:** 10.3390/cancers18132099

**Published:** 2026-06-28

**Authors:** Arezoo Modiri, Hairong Chen, Timm-Michael L. Dickfeld, Jeffrey D. Bradley, Amit Sawant

**Affiliations:** 1 Department of Radiation Oncology, University of Maryland School of Medicine, Baltimore, MD 21201, USA; hairongchen@som.umaryland.edu (H.C.); asawant@som.umaryland.edu (A.S.); 2 Cardiology, University of Maryland Medical Center, Baltimore, MD 21201, USA; tdickfel@som.umaryland.edu; 3 Department of Radiation Oncology, University of Pennsylvania, Philadelphia, PA 19104, USA; jeffrey.bradley@pennmedicine.upenn.edu

**Keywords:** radiation toxicity, cardiopulmonary system, dose response modeling, cardiac toxicity, pulmonary toxicity, heart and lung interactions

## Abstract

Thoracic radiotherapy, widely used in the treatment of mediastinal tumors, frequently exposes both the heart and lungs to ionizing radiation. While physiological interactions and shared vulnerability of the two organs have been known, heart and lung toxicities are assessed separately due to the lack of systematic studies and computational models demonstrating their interactions. For instance, based on the most updated NCCN guidelines for esophageal cancer, radiation prescriptions for preoperative, postoperative, and definitive intents are 41.4–50.4 Gy. Cardiopulmonary toxicities are well-known treatment side-effects in this disease site. Therefore, an exhaustive multi-layered list of dose-limits for lung and heart protection are recommended by NCCN guidelines. However, no combined or conditional (on the other organ’s status) dose-limits are considered. Furthermore, while recommendations for dosimetric consideration of substructures during planning are becoming more common, whole heart and whole lung doses stay the main references for cardiac and pulmonary toxicities. A growing body of pre-clinical and clinical studies has investigated the interaction between cardiac and pulmonary systems in the context of radiation-induced toxicity. However, an analysis of cardiopulmonary system’s collective response to radiation—particularly in predictive modeling, toxicity assessment, and treatment planning—is currently lacking. Here, we review the harmful interdependent interactions between the heart and lungs, especially in cancer patients and in response to treatment. Then we study the existing cardiopulmonary system models and conclude with potential strategies to collect the required parameters for models applicable to radiotherapy.

## Simple Summary

Radiation therapy in the chest area can cause toxicity in the heart and lungs. The two organs have close physiological interactions, making their health dependent on each other. However, in current radiation therapy practice, the heart and lungs are assessed for toxicity separately. Radiation oncology studies are increasingly recognizing the importance of interactions between the cardiac and pulmonary systems in the context of treatment-induced toxicity. However, a representative mathematical model of the cardiopulmonary system’s collective response to radiation is lacking. If such a model is developed, a more comprehensive understanding of cardiopulmonary system interactions during cancer therapy can be gained. Additionally, the model could be incorporated into the radiation treatment planning optimization process to further improve patient care and post-treatment quality of life. We review the harmful interactions between the heart and lungs in cancer patients and summarize potential strategies to develop models applicable to radiotherapy using existing cardiopulmonary system models.

## 1. Introduction

Cardiac toxicity from radiation therapy has been well recognized and become an active subject of research, triggering ever-evolving clinical recommendations [1,2]. Cardiovascular diseases (CVDs) play a critical role in oncology practice. On the one hand, cancer and CVDs have a two-way relationship, each worsening the other [3,4,5,6]. On the other hand, CVDs alter the clinical course in cancer therapy [7]. Velusamy et al., in their cardio-oncology State-of-the-Art review, specified that “Despite a reported 10 million deaths from cancer, there are an estimated 16.9 million cancer survivors in the US, with the number projected to increase to 22.2 million by 2030. CVD accounts for 27% of all deaths and 56% of noncancer deaths in cancer survivors” [8]. While not mainstream in clinical practice, there is a growing recognition of the importance of individual cardiac substructure responses to radiation [9,10,11,12,13,14,15,16].

Similar to the evolving understanding of cardiac response to radiotherapy, the understanding of pulmonary response to radiation therapy has grown. A simplified assumption of the lung as a parallel structure has matured to recognition of the lung’s parallel + serial structural design [14,17]. While not mainstream in clinical practice, there is a growing recognition of the importance of radiation-dose–responses of individual airways and lung sub-lobar volumes in post-treatment patient quality of life [14,17].

A way to analyze the comprehensive response to radiation from the cardiopulmonary system is yet to be found. As a partial attempt and in a slow pace, the significance of cardiac burden caused by damage to the lungs is being recognized [18]. However, whole heart and whole lung dose-limits remain the main references for cardiac and pulmonary toxicities in clinical radiation therapy [19].

**Figure 1:**
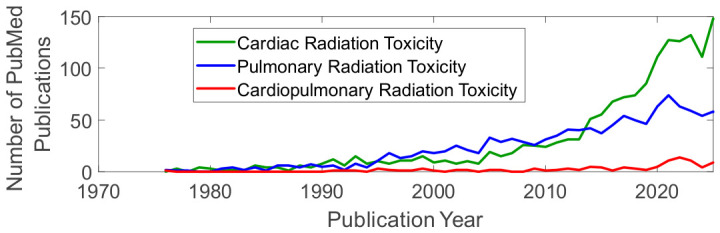
Annual publication counts in PubMed until the end of 2025. The phrases sought in title or abstract of the papers published on PubMed were 1—(cardiac toxicity or cardiotoxicity or cardio toxicity or cardiac side-effect or cardiac side effect) and (radiation therapy or radiotherapy); 2—(lung toxicity or pulmonary toxicity or lung side-effect or lung side effect or pulmonary side-effect or pulmonary side effect) and (radiation therapy or radiotherapy); and 3—cardiopulmonary and (toxicity or side-effect or side effect) and (radiation therapy or radiotherapy).

For the sake of illustration, the number of publications studying radiation-induced toxicity to the heart, to the lungs, and combined have been shown in Figure 1. The scope of our manuscript is limited to identifying systematic methods to include harmful interactions between heart and lungs in radiation treatment processes for cancer patients.

## 2. Methods and Materials

As expected, studies that discussed interactions of the heart and lungs in the context of thoracic radiotherapy were rare. Additionally, no studies were found on potential integrated cardiopulmonary system models for radiotherapy-induced toxicity assessment. Hence, our article was not structured as a systematic review but as a narrative review. We used search phrases and timelines mentioned in Figure 1 in PubMed, and then we excluded the papers investigating patient survival without assessment of any specific cardiac or pulmonary diseases or interactions. The majority of the papers shown in Figure 1 for cardiopulmonary system curve had a heavier emphasis on either heart or lungs (and not both), as discussed in the coming sections. We had to open our search scope to beyond radiotherapy in order to summarize commonplace harmful heart and lung interactions and to identify existing models potentially usable for radiotherapy.

Our goal was to present a methodological roadmap for cardiopulmonary system modeling in the context of radiotherapy toxicity. However, as a first-of-its-kind review, we find it necessary to present required background information. We start with a summary of monitoring methods in current clinical practice to establish a baseline. Then, we review published mechanistic assessments of harmful interactions between the heart and the lungs, where we also dig deeper into the heart and lung interactions in response to thoracic cancer therapy. Next, we discuss the existing cardiopulmonary system models and point out the pros and cons of these models for assessing cardiopulmonary system toxicity from radiation. In the Discussion, we outline approaches that, to our knowledge, should be followed to enable comprehensive assessment of radiation-induced cardiopulmonary toxicity.

Where needed for conveying a complete message, with the inclusion of the sources and copyright licenses, we have taken data from a figure or a table from other review manuscripts. Generative AI has been used in making some of the summary tables. Specifically, summary texts from author-chosen references were given as inputs to ChatGPT GPT-5.5 for generating a couple of tables as noted in the captions. The output was verified for accuracy by the authors. No AI-generated text appears in the manuscript body without verification.

## 3. Current Measures for Cardiopulmonary System Functionality in Cancer Patients

The following measures are well-established clinical routines, with each focusing on either a specific cardiac or specific pulmonary system functionality. For each measure, we summarize what would be understood regarding a cancer patient’s health.

- Left Ventricular Ejection Fraction (LVEF)—It assesses global systolic cardiac function and is a standard method to monitor for therapy-induced cardiotoxicity. It declines with cardiotoxic treatment [20].
- Global Longitudinal Strain (GLS)—It assesses subclinical myocardial dysfunction and is a more sensitive early indicator of cardiotoxicity than LVEF [20].
- Cardiac Biomarkers (troponin and NT-proBNP)—They assess myocardial injury and wall strain and can detect cellular cardiac injury and dysfunction during cancer therapy [21].
- Electrocardiogram (ECG)—It assesses electrical activity and rhythm, and is a standard tool for monitoring arrhythmias/QT changes in cancer patients on cardiotoxic drugs [22].
- Pulmonary Function Tests (PFTs)—They include forced expiratory volume in 1 s (FEV1), forced vital capacity (FVC), and diffusing capacity of the lung for carbon monoxide (DLCO). They assess airflow and gas exchange capacity and can detect pulmonary impairment [23].
- Peak Oxygen Consumption (VO_2_peak)—It can be assessed with Cardiopulmonary Exercise Testing (CPET). It quantifies cardiorespiratory fitness and is a predictor of functional impairment and treatment outcomes [24,25]. Anaerobic Threshold (AT) has also been considered as a replacement for VO_2_peak [26]. Another measure in this category is Ventilatory Efficiency (VE/VCO_2_ slope) [27].
- Six-Minute Walk Test (6MWT)—It can be used for the assessment of functional capacity when CPET is unavailable [24].
- Oxygen Saturation (SpO_2_)—This is routinely used in clinical practice using pulse oximetry and has potential value as a non-invasive prognostic factor in cancer patients and as a predictive factor for response to radiation therapy [28,29].
- Performance Status (ECOG and Karnofsky)—This is routinely used in clinical practice and is also a surrogate of overall cardiopulmonary reserve in oncology [30].
- Blood Pressure Monitoring—Blood pressure is monitored to assess baseline or treatment-related hypertension [31].

Note that in order to model cardiopulmonary system collectively, at the very least, these existing measures need to be performed in a time-coordinated manner, i.e., incorporating cardiac and respiratory phases.

## 4. Mechanistic Assessments of Harmful Interactions Between Heart and Lung

This section aims to provide a concise while precise description of well-identified harmful interactions between the heart and lungs.

### 4.1. Cardiorespiratory Vicious Cycles

Lung disease can have cardiac consequences such as pulmonary hypertension, pulmonary heart disease (with potential development of cor pulmonale), and an increased risk of coronary artery disease [32,33]. Although the biologic pathways linking pulmonary and cardiovascular dysfunction are not clear, chronic systemic inflammation appears to be one important underlying pathophysiologic link [34]. Association between lung function (in terms of FEV1 and FVC) and coronary artery disease (CAD) is summarized through the lenses of two different large-scale studies in Table 1 [32].

**Figure 2:**
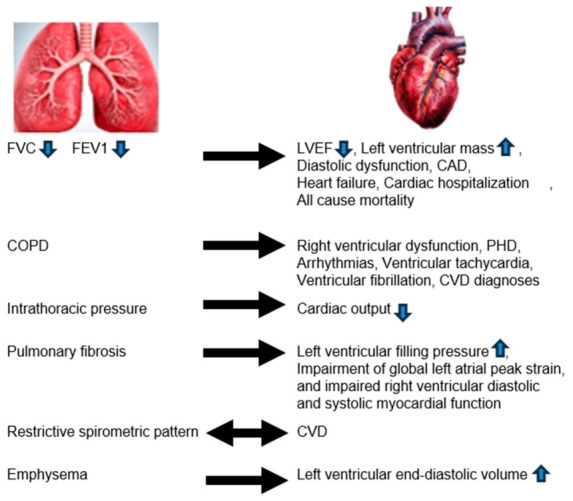
Associations between lung and heart dysfunctions based on this subsection’s literature review [32,33,34,35,36,37,38,39,40,41,42,43,44,45,46,47,48,49,50,51]. FEV1, forced expiratory volume in 1 s; FVC, forced vital capacity; CAD, coronary artery disease; PHD, pulmonary heart disease; CVD, cardiovascular disease; COPD, chronic obstructive pulmonary disease.

Table 1 studies showed that a reduction in FEV1 and FVC raised CAD risk. Decreased FVC and FEV1 were also both associated with decreased LVEF based on another study [37]. Moreover, decreased FVC was associated with increased cardiac hospitalization (hazard ratio [HR], 0.80; 95% confidence interval [CI], 0.67–0.96) even in patients with normal LVEF (HR, 0.75; 95% CI, 0.57–0.97) [37]. And in individuals with FVC < 2.75 L, decreased FVC was associated with increased all-cause mortality (HR, 0.49; 95% CI, 0.29–0.82) [37]. The first row in Figure 2 shows a simple schematic representation of these correlations.

Forfia et al. investigated airway obstruction and pulmonary heart disease (PHD) [38]. PHD refers to altered structure or function of the right ventricle occurring in association with abnormal respiratory function. Abnormal gas exchange is a fundamental underpinning of PHD, affecting pulmonary vascular, cardiac, renal, and neurohormonal systems. In the context of airway obstruction, alveolar pressure increases and can exert a compressive effect on the neighboring pulmonary vasculature. As such, with marked hyperinflation, the pulmonary vascular resistance will increase. This scenario is especially relevant during extremes of hyperinflation, as seen in patients with acute exacerbations of asthma or chronic obstructive pulmonary disease (COPD). Following the principles of ventricular–vascular coupling, the greater the degree of right ventricular dysfunction at baseline is, the greater the hemodynamic significance of any added vascular load imposed by the Starling resistor effect of airway pressure on pulmonary resistance will be [38]. Right heart failure can be caused by an increased pulmonary vascular resistance, leading to increased right heart pressures [39]. Patients with COPD have higher odds of being diagnosed with any CVD hospitalization (approximately 42% for first admission and 48% for readmission), with heart failure as the leading cause of hospitalization [40,41,42]. The second row in Figure 2 shows a simple schematic representation of these correlations.

Verhoeff et al. reviewed heart and lung interactions, with special focus on the effect of the limited amount of space the two organs share [43]. Lungs have remarkable reserve volume because under a forced condition, they can inhale and exhale 4 L of air. Interactions including external constraint to the heart, blood volume redistribution (venous return), direct ventricular interaction, and left ventricular afterload are present to one degree or another during normal respiration but can be altered largely in abnormal situations. External constraint of the heart influences cardiac output when it limits diastolic filling. As the heart and lungs lie in proximity, increased intrathoracic pressure (e.g., in patients with emphysematous lungs) increases the pressure around the heart itself. An example is the occurrence of acute tension pneumothorax as cardiac output decreases rapidly with increasing intrathoracic pressure [43]. The third and last rows in Figure 2 show simple representations of these correlations.

Sonaglioni et al. showed that left ventricular filling pressure was significantly increased in idiopathic pulmonary fibrosis patients in comparison to controls (average E/e′ ratio, 14.4 ± 3.0 vs. 9.6 ± 1.5, *p* < 0.0001), and global left atrial peak strain was significantly impaired in these patients in comparison to controls (18.4 ± 3.7% vs. 28.4 ± 5.6%, *p* < 0.0001) [44]. The likely process of ventricular interdependence in non-advanced idiopathic pulmonary fibrosis patients results in left ventricular diastolic dysfunction and secondary impairment in left ventricular—Global Longitudinal Strain and global left atrial peak strain. Other studies have shown that impaired right ventricular diastolic and systolic myocardial function were present even in idiopathic pulmonary fibrosis patients without pulmonary hypertension [45]. See Figure 2, row 4.

Ramalho et al. reviewed several trial reports focusing on the relationships of pulmonary disease and dysfunction with common incident CVD and with alterations in cardiac structure and function [34]. The studies they reviewed confirmed the abovementioned correlations [40,42,46,47,48,49,50]. Ramalho et al.’s conclusions are also added to Figure 2’s graphical summary.

### 4.2. Cardiorespiratory Interactions in Cancer Patients Receiving Thoracic Radiotherapy

Radiation therapy is a standard of care in several cancers affecting the thorax, such as lung, esophagus, and lymphatic system cancers. Technological advances and an improved understanding of disease progression and normal tissue complications have resulted in reduced cancer death rates. Low-dose computed tomography (CT) screening in high-risk populations has improved early diagnosis rates. However, cancer survivors are affected by a spectrum of side effects from their treatments [52,53,54,55]. Ghobadi et al., in their landmark rat study, irradiated the heart, lung, or both, and measured downstream functional outcomes [12]. For the first time, they showed that both lung and heart irradiation caused both cardiac and pulmonary toxicity. This finding was a demonstration of the deleterious effects of heart and lung co-irradiation [12].

Voshart et al. discussed a chain of cardiopulmonary response to radiation in a preclinical model [56]. It was shown that damage and remodeling of pulmonary vasculature led to higher right ventricle systolic pressure and right ventricle hypertrophy. In turn, these effects contributed to reduced left ventricle diastolic function. In addition, it was shown that the irradiation of the heart could cause myocardial damage, reducing diastolic function. The consequential congestion in the pulmonary vasculature caused interstitial edema, parenchymal inflammation, and fibrosis in lung tissue [56]. In their broad review, Barazzuol et al. also discussed these integrated responses [13]. Table 2 shows the cascade effect on cardiopulmonary system from radiotherapy over time.

Similarly, Wiedemann et al. studied vascular damage and vascular remodeling occurring in the time frame of several weeks after irradiation [18]. The loss of vascular endothelial cells and the consequent loss of barrier function were accompanied by the thickening of vessel walls, occlusion of small vessels, and perivascular edema. Vascular remodeling was observed after whole thorax irradiation in several studies performed in small animals [57,58]. In addition, those studies reported enhanced vascular resistance in the lung. Such increased resistance in the pulmonary vasculature led to increased pulmonary artery pressure, which in turn could induce or worsen right ventricle remodeling followed by a reduction of right ventricular performance [18].

#### Competing Risks and Risk Modeling Difficulties

Several risk factors can affect cardiopulmonary system response to radiation [2,59,60]. For example, radiation long-term side-effects have greater impact on those treated at a younger age. Another example is biological sex, as men have higher risk of cardiotoxicity compared to women. Family/personal history of CVDs, previous cancer treatments, surgery, tumor location, hypertension, hypercholesterolemia, diabetes, and smoking history are other common risk factors [2,61].

In this context, lung cancer patients have an exceptional place. The intertwined connection between cardiac and pulmonary systems makes it difficult to separate CVD symptoms from those of lung cancer, as well as to separate the effect of cancer itself from that of the treatment [62,63]. Mędrek et al. suggested that the changes in some parameters of left and right ventricular cardiac function might be a consequence of the progression of inoperable lung cancer during systemic therapy [64]. This finding is not limited to systemic therapy. Lin et al. recently conducted a mapping research that highlighted the increasing focus on the link between lung cancer and heart failure after radiotherapy [65]. Their investigated studies showed an overlapping effect related to the cardiac burden of lung cancer treatment and that of the progression of lung cancer disease. Wang et al. studied 278,418 lung cancer patients diagnosed from 1990 to 2020 [61]. They reported 12,584 CVD diagnoses within a median follow-up of 9 months (interquartile range, 3–27 months). They concluded by emphasizing “the necessity of integrating cardiovascular risk assessment and management into lung cancer treatment protocols, particularly during the first month after diagnosis and for younger or high-risk subgroups.”

An additional competing risk is COPD, common among lung cancer patients [66]. As the summary of mechanistic cascade in Section 4.1 showed, COPD is a known risk factor for CVD diagnosis and mortality [67] (Figure 2).

In the CLARITY trial (NCT04305613), a multicenter, longitudinal, prospective cohort study, it was shown that heart dose was associated with LVEF declines in 125 patients with non-small-cell lung cancer (NSCLC), with a subset experiencing clinically relevant cardiac dysfunction [68]. The fact that several studies have found significant correlation between radiation dose to the heart (or its substructures) and survival in lung cancer patients in the absence of clinical reports of CVD diagnoses for the majority of these patients points to a silent cardiac health decline [34,63]. Cardiopulmonary system management is increasingly critical, as radiation therapy is expanded to treat more centrally located tumors [69], as well as noncancer conditions such as ventricular tachycardia [70].

## 5. Cardiopulmonary System Models

**Figure A1:**
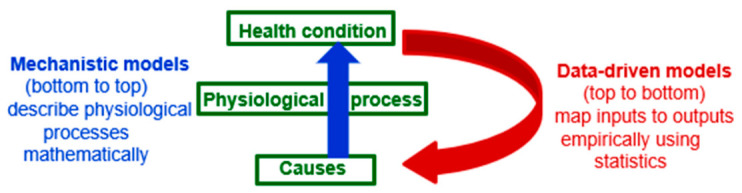
Mechanistic models describe physiological phenomena mathematically by modeling how the system works internally. On the other hand, data-driven response models map inputs to outputs empirically.

In general, mechanistic models require biologically explainable, experimentally derived knowledge of a procedure or system, whereas data-driven models rely on recognizing patterns from a sufficiently large set of existing sample cases (Figure A1). In their review, Casson et al. described how mechanistic modeling approaches have been applied to study the underlying effects of cancer treatments on the human heart [71]. They mentioned existing opportunities to combine bottom-up mechanistic models with top-down data-driven models in attempts to identify ways to stratify patients based on risk in cardio-oncology.

### 5.1. Mechanistic Models

**Figure 3:**
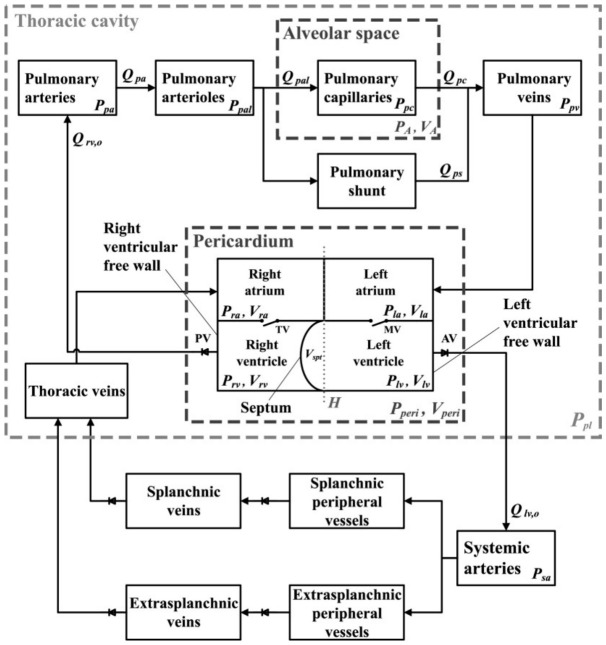
Schematic block diagram of the cardiovascular system of Karamolegkos et al.’s enhanced cardiopulmonary model. Required model parameters are as follows: P_sa_ and P_pa_, systemic and pulmonary arterial blood pressures; P_ra_ and P_la_, right and left atrial pressures; P_rv_ and P_lv_ right and left ventricular pressures; P_pal_, pulmonary arteriolar pressure; P_pc_ pulmonary capillary pressure; P_pv_, pulmonary venous pressure; P_peri_, pericardial pressure; P_pl_, pleural (intrathoracic) pressure; P_A_, alveolar pressure; V_ra_ and V_la_, right and left atrial volumes; V_rv_ and V_lv_, right and left ventricular volumes; V_spt_, septal volume; V_peri_, pericardial volume; V_A_ alveolar volume; Q_rv,o_, and Q_lv,o_, right and left ventricular output blood flows; Q_pa_, pulmonary arterial blood flow; Q_pal_, pulmonary arteriolar blood flow; Q_pc_, pulmonary capillary blood flow; Q_ps_, pulmonary shunt blood flow; MV, mitral valve; AV, aortic valve; TV, tricuspid valve; PV, pulmonary valve; H, imaginary plane defining the volumes of the septum and of the right and left ventricular free walls. This figure is copied from Figure 2 in Karamolegkos et al.’s manuscript [75] and is licensed under a Creative Commons Attribution 4.0 License.

Bai et al. generated a closed-loop electrical circuit model demonstrating correlation between lung injury severity and cardiac function [72]. In a more comprehensive manner, Albanese et al. presented an integrated mathematical model of the cardiovascular and respiratory systems and their interactions [73]. The model included heart (four chambers), systemic circulation (arteries—five peripheral and five venous compartments), pulmonary circulation (arteries, vessels, veins, and shunt), lung mechanics (larynx, trachea, bronchi, alveoli, pleural cavity, and diaphragmatic muscle), thoracic cavity, autonomic nervous system (sympathetic and vagal), local autoregulation, ischemic response of central nervous system, and gas exchange (lung and tissue), including Bohr and Haldane effects. The model was presented in two papers: one describing the model development and verification under normal physiological conditions [73], and the other describing the response under hypercapnic and hypoxic conditions [74]. Their work is quite detailed and oriented toward modeling control of breathing, gas-exchange, and general cardiopulmonary interactions under environmental or physiological perturbations (Figure 3 shows their enhanced model [75]). The relationship between inputs and outputs of each compartment is explained by a parametrized equation. Model parameters in several statuses (e.g., rest or exercise) can be found within this article or similar articles. The application of this model to assessing toxicity caused by cancer therapies, while seemingly feasible, requires specific additional parameterizations.

There are software tools at various validation levels that estimate the parameters needed for Figure 3 model from standard-of-care images using Computational Fluid Dynamics. Table A1 gives some examples of these tools [76,77,78]. These software tools also have potential in estimating patient specific heart and/or lung functionality based on blood flow measures pre- and post-radiotherapy in order to model toxicity.

### 5.2. Data-Driven Models

Table 3 summarizes some of the data-driven approaches for cardiopulmonary system modeling. While valuable, these existing models are limited to few functionality measures and cannot represent the whole cardiopulmonary system’s functionality. In addition, clinical validation through prospective studies is yet to be completed.

## 6. Discussion

Despite increasing recognition of intertwined cardiac and pulmonary burden of thoracic radiotherapy, including at the sub-organ level, cardiopulmonary system toxicity from radiation remains incompletely understood [68,84]. Physiological interdependence, shared dosimetric exposure, and improvement in predictive models with combined metrics are evidence of the need to examine cardiac and pulmonary systems together and not separately [84,85]. The ongoing NCT06410300 trial is an example of a solid step toward combined assessment of cardiopulmonary system in response to radiation. The trial’s team aims at cardiopulmonary monitoring in lung cancer patients receiving combined thoracic radiotherapy and immunotherapy. They collect cardiac troponins, brain-type natriuretic peptides, inflammatory markers (e.g., C-reactive protein), markers of oxidative stress or endothelial injury (e.g., galectin-3 or soluble ST2), PFTs (e.g., spirometry—FEV_1_ and FVC), CPET, and ECG. The team aims to associate these measures to various heart and lung radiation doses. While such studies can generate high-level data-driven models of cardiopulmonary toxicity, they will not model cardiopulmonary system interconnections.

Several refined coupled models of the cardiovascular and respiratory systems in multi-compartment designs have been developed outside the field of radiation therapy [75,86,87]. These models appear to contain the essential physiological components required for toxicity estimation in radiation oncology. However, their application to radiotherapy is challenged by the need to determine numerous parameters for each sub-organ (Figure 3). Samiento et al. presented a complexity reduction strategy for fitting and validating physiological models with a large number of parameters under the consideration of different populations and stimuli [88]. Their model was originally used for the cardiopulmonary system response prediction for healthy humans under rest and aerobic dynamic exercise conditions [89]. For radiotherapy purposes, one possible parameter estimation method is applying Computational Fluid Dynamics to standard-of-care images (Table A1). However, the current images are not respiratory and cardiac cycle specific.

Another way to perform the parameter estimation required for these existing models is to administer clinical measurements in a time-coordinated manner. Karamolegkos et al.’s model, for instance, compared to physiologically detailed integrated models, is more amenable to parameter estimation from clinically available measurements [75]. To estimate model parameters, a core set of clinical measurements should be acquired simultaneously and time-synchronized. Some of these measurements are summarized in Table 4. Several of these procedures are already part of the standard of care but are not time-synchronized or not performed at the two main points of interest (baseline pre-radiotherapy and post-radiotherapy). The current standard-of-care measurements are used selectively based on clinical need rather than as a fixed bundle. For instance, in standard lung cancer care, a routinely performed non-invasive test is spirometry, and it is typically done as a standalone Pulmonary Function Test, not synchronized with cardiac measurements. Likewise, ECG monitoring is performed independently for cardiac assessment and without simultaneous collection of respiratory flow or volume signals. Although bedside monitoring systems in some settings can record ECG and respiratory signals together, this is not part of standard-of-care workflows for radiotherapy patients. Procedures like echocardiography may incidentally capture some respiratory variation (since breathing continues during imaging), but they are not designed or recorded as synchronized cardiopulmonary time series, and respiratory signals are usually not quantified alongside cardiac outputs in a way that is usable for parameter estimation. Therefore, while pieces of the needed data exist across standard tests, they are not acquired in a way suitable for characterizing heart–lung interactions as required by the existing models.

**Figure 4:**
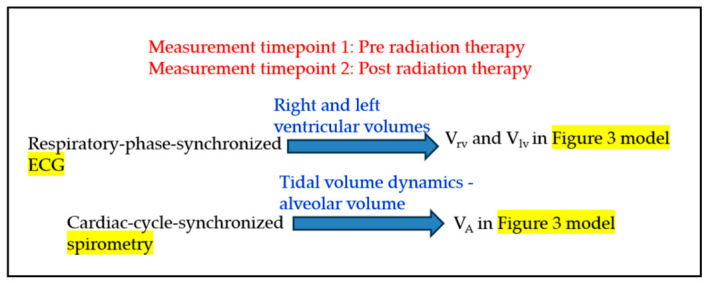
A visual summary of a potential workflow for performing the required clinical measurements (Table 4) for calculating three sample parameters in Karamolegkos et al.’s cardiopulmonary system model (Figure 3) [75]. The three sample parameters are right and left ventricular volumes and alveolar volume. The goal is to assess the changes from pre- to post-radiotherapy timepoints to predict clinical endpoints (Figure 2). The measurements will be patient-specific and under any existing competing risk (Section Competing Risks and Risk Modeling Difficulties).

In summary, the adoption of existing refined models in radiotherapy seems to be a realistic goal dependent on model parameter calculations under radiotherapy conditions and timeline. Future studies aiming in this direction need to configure their workflow to include information from respiratory and cardiac cycles within clinical measures performed before, during, and after radiotherapy. Note that the aimed types of measurements are in contrast to those summarized in Section 3, where respiratory and cardiac cycles are lost in time averaging. Hence, the new measurement timings and orders may introduce additional cost and training load to clinical practice. Figure 4 shows a potential workflow for measuring three sample parameters in Karamolegkos et al.’s cardiopulmonary system model [75].

## 7. Conclusions

Radiation oncology studies are increasingly recognizing the importance of interactions between cardiac and pulmonary systems in the context of radiation-induced toxicity. However, a representative mathematical model of the cardiopulmonary system’s collective response to radiation is lacking. There are existing detailed multi-compartment cardiopulmonary models that could potentially be used in radiotherapy. However, these models require time-resolved, coupled cardiac and respiratory signals to estimate parameters governing heart–lung interactions. To calculate patient-specific model parameters, a set of clinical measurements should be acquired simultaneously. Many of these measurements are already part of the standard of care; however, they need to be performed and analyzed in a time-synchronized manner. Once such a model is completed, it can not only give a more comprehensive picture of cardiopulmonary system interactions during cancer therapy but also enter therapy optimization process prospectively to further improve patient care and post-treatment quality of life by reducing cardiopulmonary burden of cancer therapy in a systematic way.

## Figures and Tables

**Table 1:** Statistically significant association between lung function and coronary artery disease [32,35,36]. FEV1, forced expiratory volume in 1 s; FVC, forced vital capacity; CAD, coronary artery disease; y, year; IVW, inverse variance-weighted Mendelian randomization.

Study Type	Sample Size	Measure	FEV1	FVC
**Mendelian randomization** [36]	92,749	Odds ratios for CAD and 95% confidence intervals per standard-deviation increment based on IVW	0.78 [0.62, 0.98]	—
**Rancho-Bernardo observational study** [35]	1548	Hazard ratios for ≤22-y incidence of CAD per standard-deviation increment in FEV1 or FVC	Age-adjusted for men: 0.82 [0.71, 0.93]	Age-adjusted for men: 0.82 [0.73, 0.93]
			Age-adjusted for women: 0.81 [0.71, 0.94]	Age-adjusted for women: 0.81 [0.70, 0.93]

**Table 2:** A summary of the side effects of radiotherapy (RT) on the cardiopulmonary system over time. The information is extracted from Figure 2 in Barazzuol et al.’s manuscript [13] © The Authors. Published by FEBS Press and John Wiley & Sons Ltd.

	Hours–2 Weeks Post-RT	8 Weeks–3 Months Post-RT	3 Weeks–6 Months Post-RT	≥9 Months Post-RT
**Step-by-step effects**	Loss and dysfunction of endothelial cells	Hyperproliferation of smooth muscle cells, vascular occlusion, pulmonary hypertension, alveoli acute inflammation, alveoli initial fibrosis, loss of diastolic function, enhancement of inflammation and fibrosis, risk of early cardiac failure	Alveoli chronic inflammation, alveoli function limiting fibrosis	Expansion of unaffected lung tissue, late cardiac failure
**Overall effect**	Dysfunctional endothelium	Cardiopulmonary dysfunction	Pulmonary fibrosis	Compensation and/or failure

**Table 3:** Sample data-driven models for cardiopulmonary system. DL, deep learning; ML, machine learning; SVM, support vector machine; CPET, Cardiopulmonary Exercise Testing; ECG, electrocardiogram; ICU, intensive care unit. Summary texts from author-chosen references were given as inputs to ChatGPT GPT-5.5 for generating this table.

Model/Approach	Model Type	Focus	Key Input Data or Method
**Survival model for heart failure outcomes** [79]	DL + survival analysis	CPET data	Breath-by-breath CPET features
**Random forests and gradient boosting for VO_2_ peak prediction** [80]	ML regression	Cardiopulmonary exercise physiology	Non-exercise predictor set
**Cardiac arrest prediction** [81]	ML classification model	ICU cardiac arrest risk	ECG heart rate variability features
**Hybrid lung ventilation imaging synthesis** [82]	Hybrid DL + physiological model	Lung ventilation patterns	Multi-inflation CT + modeling + DL
**Classical ML models for cardiopulmonary events (XGBoost/SVM)** [83]	Traditional ML	CPET event prediction	Exercise and clinical predictors

**Table 4:** A list of measurements that are required to provide some of the cardiopulmonary system parameters based on Karamolegkos et al.’s model [75]. Summary texts from author-chosen references were given as inputs to ChatGPT GPT-5.5 for generating this table.

Procedure Summary	Cardiopulmonary System Parameter
Transthoracic echocardiography using computer-aided guidance	Ventricular elastance, stroke volume, ventricular–arterial coupling
Ultrasonography of the heart and aorta	Global cardiac geometry, ventricular compliance, aortic impedance
Standard echocardiography of the heart	Left/right ventricular volumes, ejection fraction, baseline elastance
Cardiac magnetic resonance imaging	Structural parameters (ventricular volume, wall mass, compliance constants)
External cardiac function monitoring	Time-varying cardiac output, stroke volume dynamics, coupling gain
Ambulatory echocardiography monitoring	Heart rate variability, autonomic modulation parameters, chronotropic control gains
Cardiac stress monitoring	Reserve contractility, load-dependent elastance, autonomic response sensitivity
Spirometry (lung capacity)	Lung compliance, tidal volume dynamics, respiratory system elastance
Spirometry (airflow)	Airway resistance, respiratory time constants, flow–pressure relationship
Respiratory rate measurement	Respiratory rhythm generator parameters, cardiopulmonary phase coupling frequency

## Data Availability

No new data were created or analyzed in this study.

## Abbreviations

The following abbreviations are used in this manuscript:

AT	Anaerobic Threshold
CAD	Coronary artery disease
CI	Confidence interval
COPD	Chronic obstructive pulmonary disease
CPET	Cardiopulmonary Exercise Testing
CT	Computed tomography
CVD	Cardiovascular disease
DL	Deep learning
DLCO	Diffusing Capacity of the Lung for Carbon Monoxide
ECG	Electrocardiogram
ECOG	Eastern Cooperative Oncology Group
FEV_1_	Forced expiratory volume in 1 second
FVC	Forced vital capacity
GLS	Global Longitudinal Strain
HR	Hazard ratio
ICU	Intensive care unit
IVW	Inverse variance-weighted
LVEF	Left Ventricular Ejection Fraction
ML	Machine learning
MR	Mendelian randomization
MRI	Magnetic resonance imaging
6MWT	Six-Minute Walk Test
NCCN	National Comprehensive Cancer Network
NT-proBNP	N-terminal pro-B-type natriuretic peptide
OR	Odds ratio
PHD	Pulmonary heart disease
PFT	Pulmonary Function Test
RT	Radiotherapy
SPO_2_	Oxygen Saturation
SVM	Support vector machine
VE	Ventilatory Efficiency
VF	Ventricular Fibrillation
VO_2peak_	Peak Oxygen Consumption
VT	Ventricular tachycardia
y	Year

## Appendix A

**Table A1:** Sample open-source software tools capable of calculating the required parameters in Figure 3 directly or indirectly.

Software	Input
SimVascular [76] December 2025 Release	CT Angiography (CTA), MR Angiography (MRA), Phase-Contrast MRI
CRIMSON [78] June 2025 Release	CTA, MRA, contrast and non-contrast CT
VaSP [77] Version 3	Vessel masks extracted from images
